# Development and Application of Polymer Scaffolds

**DOI:** 10.3390/polym17162260

**Published:** 2025-08-21

**Authors:** Wang Guo

**Affiliations:** 1 State Key Laboratory of Featured Metal Materials and Life-Cycle Safety for Composite Structures, School of Mechanical Engineering, Guangxi University, Nanning 530004, China; guowang@gxu.edu.cn or drwangguo@outlook.com; 2 Institute of Laser Intelligent Manufacturing and Precision Processing, Guangxi Key Laboratory of Manufacturing System and Advanced Manufacturing Technology, School of Mechanical Engineering, Guangxi University, Nanning 530004, China

## 1. The Evolving Landscape of Polymer Scaffolds in Regenerative Medicine

It is with great pleasure that we introduce this Special Issue of *Polymers*, entitled “Development and Application of Polymer Scaffolds” (https://www.mdpi.com/journal/polymers/special_issues/TS7AA7MSI8 accessed on 18 August 2025). This collection was conceived to capture the dynamic and rapidly advancing research in the design, fabrication, functionalization, and application of polymer-based scaffolds, which stand at the forefront of tissue engineering and regenerative medicine.

Polymer scaffolds have become indispensable tools in the quest to repair and regenerate damaged tissues. They serve as temporary, three-dimensional templates that mimic the native extracellular matrix (ECM), providing the necessary structural support and guidance for cells to adhere, proliferate, differentiate, and ultimately form new, functional tissue. The widespread adoption of polymers for this purpose is rooted in their remarkable versatility; their properties, including biocompatibility, biodegradability, mechanical strength, and processability, can be precisely tailored to meet the demanding requirements of diverse biological environments.

The field has undergone a significant evolution. Early research primarily focused on creating scaffolds that were biocompatible, possessed an interconnected porous architecture to facilitate nutrient and waste transport, and degraded at a rate commensurate with new tissue formation. While these foundational principles remain critical, the contemporary paradigm has shifted towards the development of “bio-instructive” or “functional” scaffolds. These advanced constructs are no longer passive supports but are engineered to be active participants in the regenerative cascade. They can present specific biological cues to direct cell fate, release therapeutic agents in a controlled manner, or respond dynamically to physiological stimuli, thereby orchestrating a more efficient and targeted healing response.

This Special Issue brings together a collection of 14 peer-reviewed articles that exemplify this paradigm shift. The compilation features 11 original research articles, two comprehensive reviews, and one systematic review, reflecting a healthy balance between novel discovery and the critical synthesis of existing knowledge. The composition of this collection itself speaks to the maturation of the polymer scaffold field. While novel, highly specialized research continues to push the frontiers of what is possible, the inclusion of systematic reviews on topics such as 3D printing in prosthodontics and hydrogels for gingival regeneration indicates that certain sub-domains have amassed a sufficient body of literature to identify established trends and define clear future directions—a hallmark of a maturing scientific discipline. This collection, contributed by research groups from around the globe, offers a comprehensive snapshot of the current State of the Art and is intended to serve as a valuable resource for the scientific community. The global and collaborative nature of this research is highlighted by the diverse origins of the contributions, as summarized in Table 1.

## 2. Highlights of the Special Issue: A Thematic Overview of Contributions

The 14 articles published in this Special Issue cover a broad spectrum of research, from fundamental materials science to application-driven engineering. For ease of navigation, the contributions are summarized in Table 2 before being discussed in thematic detail.

### 2.1. Innovations in Scaffold Fabrication and Structural Control

Control over a scaffold’s internal architecture is paramount, as it directly influences cell infiltration, nutrient transport, and mechanical performance. The contributions in this Special Issue highlight two parallel streams of innovation in scaffold manufacturing. On one hand, established techniques are being refined for greater precision and reliability, which is essential for translating laboratory successes into consistent products. On the other hand, advanced digital fabrication methods are being leveraged to create structures of unprecedented complexity and personalization.

Representing the first stream, Song et al. [2] address a fundamental challenge in a classic fabrication method: the stochastic nature of ice nucleation during freeze-drying. By introducing low-frequency ultrasound, they demonstrate the ability to trigger nucleation on demand in collagen slurries. This novel process enables precise control over the resulting pore size and distribution, significantly improving the structural reproducibility of scaffolds—a critical step towards reliable, large-scale manufacturing.

In the second stream, the review by Dimitrova et al. [12] provides a comprehensive overview of how additive manufacturing (AM), or 3D printing, is revolutionizing the field of prosthodontics. This work underscores the transformative power of AM to fabricate patient-specific implants and devices with complex geometries that are unattainable with conventional methods. This theme resonates strongly within the broader tissue engineering field, where 3D printing is increasingly used to create anatomically accurate constructs. Together, these papers show that progress is advancing on two crucial fronts: making established, cost-effective techniques more precise and making complex, digital techniques more accessible and clinically relevant.

### 2.2. Functionalization of Scaffolds for Enhanced Biological Performance

A central theme of this Special Issue is the strategic functionalization of scaffolds to imbue them with bioactive properties that actively direct the healing process. The works collected here demonstrate a sophisticated design philosophy, where additives are selected not just to add a single feature, but to solve a constellation of interrelated material and biological challenges.

A compelling example of this integrated approach is presented by Guo et al. [3]. Polylactic acid (PLA) is a widely used biomaterial, but it suffers from several interconnected drawbacks: its slow degradation produces acidic byproducts that can cause inflammation, and its mechanical properties are often insufficient for load-bearing applications. By incorporating magnesium hydroxide (Mg(OH)_2_) nanoparticles into 3D-printed PLA scaffolds, the authors address all these issues simultaneously. The alkaline nature of Mg(OH)_2_ neutralizes the acidic degradation products, which in turn accelerates the hydrolysis of PLA for a more favorable degradation profile. Furthermore, the released magnesium ions (Mg^2+^) are known to be osteoinductive, and the nanoparticles act as a reinforcing agent, improving the scaffold’s mechanical strength. This work exemplifies a shift from single-feature enhancement to integrated system optimization.

Post-implantation infection is a major cause of failure for biomedical devices. Two articles in this Special Issue tackle this challenge from different angles. Menotti et al. [6] carefully tune the concentration of silver in poly(ε-caprolactone)/biphasic calcium phosphate (PCL/BCP) scaffolds. They identify a therapeutic window that provides potent antibacterial activity against common pathogens while remaining non-toxic to bone cells, highlighting the delicate balance required for cytocompatible functionalization. Approaching the problem from a molecular level, To et al. [7] report the design of potent and salt-insensitive antimicrobial branched peptides. This work offers a highly specific and sophisticated biological alternative to traditional antimicrobial agents like metallic ions.

Finally, pushing towards the development of “smart” scaffolds, Botvin et al. [8] fabricate magnetoactive scaffolds by incorporating iron oxide (Fe_3_O_4_) nanoparticles into electrospun polymer fibers. Such materials have the potential for remotely triggered drug release, mechanical or electrical stimulation of cells, or localized hyperthermia therapy, representing a significant step towards dynamic, responsive regenerative devices.

### 2.3. Tailored Scaffolds for Specific Tissue Engineering Applications

The diversity of tissues in the human body necessitates scaffold designs that are meticulously tailored to specific biological and mechanical requirements. The articles in this collection showcase this application-driven approach, reflecting an evolution in the concept of bio-inspiration. The field is moving beyond simply mimicking the static physical structure of the ECM to designing scaffolds that actively support and guide the unique biological processes essential for the regeneration of a specific tissue.

For nerve regeneration, Gutiérrez et al. [1] report the development of a nerve guide conduit fabricated from natural polymers—cellulose acetate and soy protein. Their comprehensive characterization focuses on properties critical for peripheral nerve repair, including suturability, flexibility, porosity conducive to Schwann cell migration, and excellent biocompatibility.

In the realm of corneal repair, Zdraveva et al. [9] address a key failure mode: abnormal blood vessel growth (neovascularization) into the transparent cornea. Their work presents a dual-function electrospun PCL scaffold. It not only provides a supportive substrate for limbal stem cells, but also delivers an anti-vascular endothelial growth factor (anti-VEGF) drug in a sustained manner. This design actively intervenes in a detrimental biological process, showcasing a functional approach to bio-inspiration that goes beyond simple structural mimicry.

For bone and cartilage regeneration, several innovative strategies are presented. Pereira et al. [11] demonstrate remarkable resourcefulness by using cuttlefish bone as a natural, sustainable source for BCP scaffolds. These marine-derived scaffolds are then coated with a novel poly(ester urea), which is shown to significantly enhance the osteogenic differentiation of human mesenchymal stromal cells. The state of the field for challenging tissues is synthesized in two key reviews. Kováč et al. [13] provide a mini-review on injectable and bioadhesive hydrogels for cartilage repair, highlighting the critical role of adhesion in ensuring scaffold integration and function. Concurrently, Hutomo et al. [14] present a systematic review of the in vitro evidence for hydrogel-based biomaterials in gingival regeneration, consolidating the current knowledge base for this important application in periodontics.

### 2.4. Advancing Drug Delivery and Material Degradation Studies

The final theme focuses on the dynamic nature of scaffolds, either as controlled release platforms or through their own degradation and resorption over time. The inclusion of fundamental materials science studies alongside application-focused work highlights a mature understanding within the field: successful clinical translation is built upon a rigorous, quantitative understanding of underlying material behaviors.

Alassaf et al. [4] develop a novel copolymer system specifically as a drug carrier. By creating a solid solution of the hydrophobic drug Mevacor within a poly(vinyl acetate-co-2-hydroxyethyl methacrylate) matrix, they significantly enhance the drug’s solubility and achieve controlled release profiles. This work underscores the potential of polymer scaffolds to address longstanding pharmaceutical challenges.

In a crucial contribution to the foundational knowledge, Deshpande et al. [5] conduct a meticulous long-term study on the in vitro degradation of PCL multifilament yarn under physiological conditions. While not a cell-based study, this work provides indispensable data for the design of any load-bearing or long-term implant. Accurately predicting the rate at which a scaffold loses its mechanical integrity and is resorbed by the body is essential for ensuring its function and preventing clinical complications. Such foundational studies are the bedrock upon which successful regenerative therapies are built.

Similarly, the work by Sangkatip et al. [10] on gelatin/Na_2_Ti_3_O_7_ nanocomposite scaffolds focuses on the systematic optimization of the material’s composition to achieve desirable mechanical properties and swelling behavior. This detailed characterization is a vital prerequisite for more advanced applications, such as controlled drug delivery, where the degree of swelling directly dictates the rate of drug diffusion and release.

## 3. Concluding Remarks and Future Perspectives

The 14 articles gathered in this Special Issue collectively paint a vibrant picture of a field characterized by rapid innovation and increasing sophistication. The overarching trend is the clear evolution of polymer scaffolds from passive, structural templates into active, multifunctional, and bio-instructive systems designed to orchestrate complex biological responses.

Building upon the excellent work presented here, several exciting frontiers are emerging. The multi-functionalization demonstrated by Guo et al. [3] and the stimuli-responsive capabilities shown by Botvin et al. [8] point towards a future of “smart” scaffolds with integrated diagnostic and therapeutic functions. One can envision future implants that sense the local biochemical environment (e.g., pH, inflammatory markers) and respond by delivering appropriate therapeutic agents on demand. The natural progression from 3D printing, as reviewed by Dimitrova et al., [12] leads to 4D bioprinting, where printed constructs can change their shape, properties, or function over time in response to stimuli. This could enable the fabrication of self-assembling devices or scaffolds that dynamically soften as new tissue forms, better matching the mechanical environment of regeneration. Furthermore, the development of advanced materials, such as the novel poly(ester urea) used by Pereira et al., [11] will continue to be a driving force. The future likely lies in hybrid bio-inks that combine the bioactivity of natural polymers with the processability and mechanical robustness of synthetic polymers, enabling the direct printing of cell-laden, living tissue constructs.

Despite this immense promise, significant hurdles remain on the path to widespread clinical translation. Key challenges include developing robust and scalable manufacturing processes, navigating complex and costly regulatory pathways, and conducting the large-scale, long-term clinical trials required to definitively prove safety and efficacy.

In closing, we extend our sincere gratitude to all the authors for their outstanding contributions, to the many expert reviewers for their diligent and constructive feedback, and to the editorial team at *Polymers* for their invaluable support in bringing this Special Issue to fruition. We hope that this collection will serve as both a valuable resource and a source of inspiration for researchers, clinicians, and engineers as we continue to advance the development and application of polymer scaffolds for the benefit of human health.

## Figures and Tables

**Table 1:** Statistical overview of the contributions to the Special Issue.

Statistic	Count
Total Authors	98
Contributing Institutions (First Level)	24
Contributing Countries	17

**Table 2:** Summary of the articles published in the Special Issue “Development and Application of Polymer Scaffolds” (Papers ordered by publication date, most recent first).

Ref.	First Author	Corresponding Author	First Affiliation (Institution, Country)	Title	Article Type	Key Focus/Contribution
[1]	Gutiérrez, B.	Dias, F.J.	Universidad de La Frontera, Chile	Comprehensive Development of a Cellulose Acetate and Soy Protein-Based Scaffold for Nerve Regeneration	Article	Fabrication and biocompatibility of a natural polymer-based conduit for peripheral nerve repair.
[2]	Song, X.	Cameron, R.E.	University of Cambridge, UK	Controlling the Architecture of Freeze-Dried Collagen Scaffolds with Ultrasound-Induced Nucleation	Article	A novel processing technique to control pore architecture and improve reproducibility in collagen scaffolds.
[3]	Guo, W.	Guo, W.; You, H.; Long, Y.	Guangxi University, China	Magnesium Hydroxide as a Versatile Nanofiller for 3D-Printed PLA Bone Scaffolds	Article	Multifunctional nanofiller to simultaneously improve mechanical, degradation, and osteogenic properties of PLA.
[4]	Alassaf, M.	Aouak, T.	Qassim University, Saudi Arabia	Mevacor/Poly(vinyl acetate/2-hydroxyethyl methacrylate) as Solid Solution: Preparation, Solubility Enhancement and Drug Delivery	Article	A novel copolymer system for enhancing the solubility and controlled delivery of a hydrophobic drug.
[5]	Deshpande, M.V.	Deshpande, M.V.; King, M.W.	North Carolina State University, USA	Degradation of Poly(ε-caprolactone) Resorbable Multifilament Yarn under Physiological Conditions	Article	In-depth analysis of PCL yarn degradation, providing crucial data for scaffold design.
[6]	Menotti, F.	Palmero, P.; Allizond, V.	Politecnico di Torino, Italy	Tuning of Silver Content on the Antibacterial and Biological Properties of Poly(ε-caprolactone)/Biphasic Calcium Phosphate 3D-Scaffolds for Bone Tissue Engineering	Article	Functionalization of bone scaffolds with silver to impart antibacterial properties without compromising cytocompatibility.
[7]	To, J.	Tam, J.P.	Nanyang Technological University, Singapore	Design of Potent and Salt-Insensitive Antimicrobial Branched Peptides	Article	A molecular design approach to creating highly effective antimicrobial peptides for biomaterial functionalization.
[8]	Botvin, V.	Botvin, V.; Surmenev, R.	Tomsk Polytechnic University, Russia	Effect of Fe_3_O_4_ Nanoparticles Modified by Citric and Oleic Acids on the Physicochemical and Magnetic Properties of Hybrid Electrospun P(VDF-TrFE) Scaffolds	Article	Fabrication of magnetoactive scaffolds for potential applications in stimuli-responsive tissue engineering.
[9]	Zdraveva, E.	Zdraveva, E.; Mijovic, B.	University of Zagreb, Croatia	The Reliability of PCL/Anti-VEGF Electrospun Scaffolds to Support Limbal Stem Cells for Corneal Repair	Article	A drug-eluting scaffold designed to prevent neovascularization and support stem cells for corneal regeneration.
[10]	Sangkatip, R.	Sriseubsai, W.	King Mongkut’s University of Technology North Bangkok, Thailand	Gelatin/Na_2_Ti_3_O_7_ Nanocomposite Scaffolds: Mechanical Properties and Characterization for Tissue Engineering Applications	Article	Development of a nanocomposite hydrogel with optimized mechanical and swelling properties.
[11]	Pereira, P.	Coelho, J.F.J.; Fonseca, A.C.	University of Coimbra, Portugal	In Vitro Evaluation of Biphasic Calcium Phosphate Scaffolds Derived from Cuttlefish Bone Coated with Poly(ester urea): for Bone Tissue Regeneration	Article	Use of a marine-derived biomaterial coated with a novel polymer to enhance osteogenic differentiation of MSCs.
[12]	Dimitrova, M.	Dimitrova, M.	Medical University of Plovdiv, Bulgaria	Recent Advances in 3D Printing of Polymers for Application in Prosthodontics	Review	A comprehensive review of the State of the Art in 3D printing for dental prosthetic applications.
[13]	Kováč, J.	Žiaran, S.	Comenius University, Slovakia	Bioadhesive and Injectable Hydrogels and Their Correlation with Mesenchymal Stem Cells Differentiation for Cartilage Repair: A Mini-Review	Review	A focused review on the critical role of bioadhesion in injectable hydrogels for cartilage regeneration.
[14]	Hutomo, D.I.	Amir, L.	Universitas Indonesia, Indonesia	Hydrogel-Based Biomaterial as a Scaffold for Gingival Regeneration: A Systematic Review of In Vitro Studies	Systematic Review	A systematic analysis of the literature on hydrogel scaffolds for periodontal soft tissue engineering.
